# Validating the Doctoral and Academic Writing in Nursing, Midwifery and Allied Health Profession Survey Questionnaire for Writing Group Interventions

**DOI:** 10.1111/jan.70290

**Published:** 2025-10-27

**Authors:** Marion Ann Waite, Mary Deane, Heather Callaghan, Helen Aveyard, Karen Lascelles

**Affiliations:** ^1^ Oxford School of Nursing and Midwifery Oxford Brookes University Oxford UK; ^2^ Centre for Academic Development Oxford Brookes University Oxford UK

**Keywords:** academic writing development, doctoral education, writing groups, writing methodology, writing research

## Abstract

**Aims:**

Despite extensive research on doctoral education, reliable tools to measure how writers' development relates to participation in social interventions such as writing groups are lacking. To address this, we conducted a study to create and evaluate a measurement tool for assessing the impact of writing group interventions on writers' development.

**Design:**

This methodology paper reports on the design, content validity, and evaluation of a new survey tool: the Doctoral and Academic Writing in Nursing, Midwifery, and Allied Health Professional writing questionnaire (DAWNMAHP).

**Methods:**

We created a pool of 39 items based on empirical articles from SCOPUS, ERIC, BEI, ZETOC, CINAHL, EBHOST, and PsycINFO, our experience, and stakeholder consultations. After a content validity assessment by writing experts, we revised the pool to 44 items in five domains. Finally, we tested it on doctoral writing workshop attendees using factor analysis, Pearson correlations, and Cronbach's Alpha evaluation.

**Results:**

Thirty‐six participants completed the DAWNMAHP survey tool: 22 doctoral students, seven early‐career researchers, and seven participants on a designated pre‐doctoral pathway. Cronbach's Alpha evaluation demonstrated good reliability (*α* > 0.70) for all five factors. This sample was deemed moderately sufficient (KMO = 0.579), and the items were loaded onto the five factors with all items' factor loadings > 0.5 through principal component analysis.

**Conclusion:**

DAWNMAHP is a novel, reliable tool that measures the impact of writing group interventions on an individual writer's development concerning time management, the writing process, identity, social domains, and relational agency.

**Implications for the Profession:**

Conducting pre‐ and post‐writing group intervention tests and recruiting larger sample sizes is essential to further developing DAWNMAHP. It is a rigorous tool for researching the benefits of writing group interventions. Furthermore, DAWNMAHP is an effective assessment and measurement tool, making a novel contribution to research into doctoral education.

**Patient or Public Contribution:**

No patient or public involvement was necessary at the validation stage of the DAWNMAHP tool.

**Summary**

- What problem did the study address?
  ○ Writing a doctorate is a complex process requiring stamina, resilience, and advanced knowledge. It is emotionally demanding and time‐consuming.
  ○ While writing groups can offer practical and psychological support, robust research instruments are lacking to assess how these groups benefit Nursing, Midwifery, and Allied Health Professionals (NMAHPs).
- What were the main findings?
  ○ DAWNMAHP is a reliable and valid tool for measuring writing development within writing group interventions.
  ○ An additional finding suggests that NMAHP doctoral writers could utilise DAWNMAHP as a learning resource, as it enhances their self‐awareness regarding their writing development.
- Where and on whom will the research have an impact?
  ○ The DAWNMAHP tool will impact writing group intervention facilitators who aim to examine their effectiveness in developing doctoral writing for NMAHPs.
  ○ Furthermore, the DAWNHAHP tool will benefit doctoral and early career researchers in evaluating their writing processes.

## 1 Introduction

Despite a wealth of research on approaches to supporting doctoral writers to navigate thesis writing (Calle‐Arango and Ávila Reyes 2023; Ciampa and Wolfe 2023; Inouye and McAlpine 2023; Kamler and Thomson 2004), there is a gap in the literature concerning the reliability, validity, and trustworthiness of research into writing group interventions in the nursing, midwifery, allied health, and social professional (NMAHP) fields. This highlights a gap in the broader pedagogical literature on writing group interventions, demonstrating their under‐research and lack of theoretical foundation (Aitchison and Guerin 2014; Badenhorst et al. 2015).

This paper aims to report the content validity, reliability, and validity evaluation of a new research instrument to measure the relationship between academic research writing development and participation in writing group interventions for doctoral and postdoctoral NMAHPs, such as writing workshops, groups, and retreats. The Doctoral and Academic Writing in Nursing, Midwifery, and Allied Health Professional writing questionnaire (DAWNMAHP) is a critical tool for research to develop valid and reliable methods for assessing NMAHP writers' development at the doctoral and postdoctoral level to create an evidence base for writing group interventions. The tool aims to measure the relationship between writing group interventions and doctoral and academic writing development based on a writer's self‐assessment of time management, writing identity development, awareness of the writing process, social dimensions of writing development, and developing agency as a writer.

## 2 Background

Doctoral writing is a complex, time‐consuming, and formative process that turns novices into recognised experts within a scholarly community (Kamler and Thomson 2004). The role of writing evolves during the doctoral journey from a means of demonstrating an understanding of existing ideas to a vehicle for constructing and disseminating original insights (Inouye and McAlpine 2023). Developmental changes include the need for individuals to understand the epistemic functions of writing, become aware of how writing can create a dialogue between the reader and the writer, develop a writer's voice, align with or counter the values or evidence of other authors, and understand how texts operate to serve and maintain disciplinary communities, including the appropriate genre (Castelló et al. 2012).

Doctoral writing is underpinned by feedback from supervisors and other scholars, who help candidates master disciplinary genres and rework ideas to develop their scholarly voice (Ciampa and Wolfe 2023). The completed doctoral thesis displays a candidate's critical thinking, problem‐solving, and innovation abilities (Calle‐Arango and Ávila Reyes 2023). The doctoral writing process thus serves as a form of apprenticeship, conferring expertise and authority to candidates upon completion.

In the UK and globally, enhancing the research skills of NMAHPs is essential. This effort, backed by policy directives and funding, recognises NMAHPs' vital research roles in positively impacting patient outcomes (Henshall et al. 2021; Jones and Keenan 2021). Consequently, a doctoral degree can be essential to NMAHP's professional development. Internationally, there is more than one doctoral pathway for NMAHPs. There are three types of doctorates: the PhD, which involves a research project culminating in a thesis of up to 100,000 words; the doctorate by publication, where the student submits published work and a supporting statement; and the professional doctorate (PD), which includes a taught component and a smaller independent research project than the other programmes. There is a Doctor of Nursing Practice (DNP) in the USA for nursing leaders focusing on patient outcomes and translational research.

NMAHP doctoral studies are commonly undertaken part‐time with an estimated completion of four to 8 years (Chapman et al. 2024) alongside employment and other life responsibilities. Chapman et al. (2024) argue that many variables can influence completion and progression, such as developing self‐efficacy as a researcher and needing to maintain clinical practice for financial and professional reasons. In addition to composing the thesis or doctoral dissertation, doctoral writers frequently produce other writing outputs, such as peer‐reviewed publications and conference abstracts, in collaboration with their supervisory teams, which also serve their formative development as doctoral writers.

Writing group interventions foster the formative processes of doctoral writing and are frequently implemented and reported on for NMAHPs. Writing group interventions potentially impact academic writing development and consequent completion and progression. A ‘writing group’ refers to situations where more than two people collaborate to write sustainably (Aitchison and Guerin 2014). The approach is profoundly social and embedded in practice. Murray and Newton (2009) outline the features of a structured writing group intervention, including support for participants beyond providing space and time to write. The aims are to offer academic writers a supportive and organised environment, fostering collaboration, feedback, and personal growth in writing practices. Key elements include goal‐setting, forming a community of practice, participating in peer‐review activities, time management, writing identity development, and activities to sustain changes following the intervention.

### 2.1 Literature Review

We undertook a comprehensive literature search and review of empirical studies of writing group interventions for the NMAHP disciplines to understand the relationship between writing interventions and doctoral and academic writing development. Using keywords that included Academic research writ* development* OR literac* AND writ* group* OR writ* retreat* OR writ* conference* OR writ* mentor*, we searched SCOPUS, ERIC, BEI, ZETOC, CINAHL, EBHOST, and PsycINFO.

We found that studies of writing group interventions for NMAHPs have focused on participants' summative outcomes through descriptive evaluations or qualitative studies. Irrespective of the methodology, productivity measured by publication outputs is the most widely reported outcome, followed by cost–benefit analyses of implementing writing group interventions (Dwyer et al. 2015; D. Jackson 2009). These perspectives are understandable, given that scholarly output is a key performance indicator in higher education. However, productivity as a research focus overlooks writing development's formative, temporal, cultural, and social nature. Johnson et al. (2017) state that a sole focus on writing group interventions as a means of production marginalises broader social and emotional discourses.

A knowledge gap exists concerning the formative development of doctoral writing skills among NMAHPs participating in writing group interventions. The knowledge gap echoes a call relating to the medical disciplines, which shows that despite many reported writing group interventions for medical academic writing, there is a lack of validated tools to measure their benefits (Astaneh, Abdullah, et al. 2024). Therefore, this paper aims to report on the content validity, reliability, and validity evaluation of a new research instrument to measure writing development within the context of writing group interventions for NMAHPs. The paper presents findings based on recommendations for demonstrating instrument content and generating items, informed by concept definition and formation, expert insights, and piloting with the target population (Almanasreh et al. 2019; Lynn 1986).

### 2.2 Developing Conceptual and Operational Definitions

The literature review included a critical appraisal and synthesis of empirical literature on writing group interventions for NMAHPs at the graduate level. We then integrated the theoretical literature on scholarly writing and human development to identify the following conceptual and operational definitions.

#### 2.2.1 Time Management

A strong theme emerging from reported writing group interventions is the value of protecting and legitimising time during a writing group intervention away from clinical demands (Bell and Murray 2021; Bonnamy et al. 2024; Garside et al. 2015; MacLeod et al. 2012; Murray 2014) and how protected time leads to demonstrable outputs (Bell and Murray 2021; Brandon et al. 2015; Dwyer et al. 2015; Henshall and Lewin 2022; D. Jackson 2009). Many authors are also concerned with evaluating how participation in a writing group impacts self‐efficacy and time management following an intervention such as goal setting and maintaining regular writing habits (R. Murray 2012, 2014; MacLeod et al. 2012; McLellan et al. 2024) and avoiding procrastination (Harne‐Britner and Leaver 2023).

Time management means setting clear goals, prioritizing tasks, creating an action plan with times and dates, concentrating on one task at a time, eliminating distractions, and using appropriate tools (P. Jackson 1968). Time management practices and techniques employed in writing group interventions include the Pomodoro technique (Sword 2012; Cirillo 2006), which is a method that helps writers break down their work into manageable intervals (typically 25 min of focused work followed by a short break). Cirillo argues that this technique can improve focus, reduce burnout, and make long writing tasks seem less daunting.

#### 2.2.2 Awareness of the Writing Process

The literature shows that writing group intervention activities explicitly focus on the writing process, including writing competencies such as, grammar (Guerin et al. 2013), sentence construction (Harne‐Britner and Leaver 2023), composition, the flow of the argument (Dwyer et al. 2015; Guerin et al. 2013; D. Jackson 2009; Kulage et al. 2020; Kulage and Larson 2016; Sanderson et al. 2012; Stone et al. 2010; Sun et al. 2019), readability, and knowledge of the writing process (Boquet et al. 2017; Weaver et al. 2014). Furthermore, selecting and targeting a journal (Dhakal and Tornwall 2020; Dwyer et al. 2015) is also important. Some studies consider how participants develop self‐awareness of the formative aspects of writing processes (Boquet et al. 2017; Harne‐Britner and Leaver 2023).

Writing process models influence practitioners and researchers who implement and investigate writing group interventions in higher education (Haas 2010). Writing process models (Bereiter and Scardamalia 1987; Elbow 1999; Flower and Hayes 1981; Kellogg 2008; D. M. Murray 1997) emphasize the cognitive aspects of writing, viewing it as a goal‐directed activity supported by sub‐goals that evolve through the writing experience. Three key components are involved: the task environment (external factors), a writer's long‐term memory (knowledge of the topic and audience), and the writing processes used to solve rhetorical challenges.

Bereiter and Scardamalia (1987) propose two models of the writing process: Knowledge‐telling, which is common among novice writers, and knowledge‐transforming. D. M. Murray (1997) describes a non‐linear approach with three interconnected stages: rehearsing, drafting, and revising, highlighting the need for ongoing evaluation and collaboration between writers and instructors. Sharing writing orally allows a writer's voice to be heard while also requiring introspection.

Kellogg (2008) builds on the notion of writing process models by comparing writing development to developing expertise in complex fields, suggesting that mastering macrostructure writing can take decades. Kellogg introduced an advanced stage called knowledge‐crafting, where writers balance planning, structuring, and reviewing their ideas. Additionally, understanding the text's message and considering the audience can create significant cognitive demands, making writing both a complex and spontaneous process.

Some authors consider writing process models to be limited to the individual. Hyland (2008) and Smagorinsky (2011) critiques concern an overemphasis on psychological factors while overlooking how context affects writing development. Similarly, Gere (1987) critiques writing process models concerning writing group interventions because the product and process dichotomy does not address the social aspects of writing by assuming that writers work alone and that writers' thoughts are generated internally. Instead, the advantages of examining the writing process within a socially enriched task environment are contended (Kellogg 2008; Smagorinsky 2011), influenced by Vygotsky's (1997) perspectives on writing development. Smagorinsky (2011) argues that a Vygotskian view enhances the environment by considering writing as a tool. It is a tool that empowers people to find meaning by making their thoughts available, which can be both well‐formed and rudimentary. Such a view exposes two tensions based upon the mutually enhancing aspects of speech communication: the sign function of writing and how it represents meaning and the tool function of how writing acts on the world. Thus, signs and tools can be a process and a product. This perspective aligns with Bazerman's (2012) view on the importance of exposing the relationship between writing and thinking, specifically writing as a developmental process to refine thinking, argument, and concept development.

A gap in the literature concerns the potential pros and cons of generative Artificial Intelligence (AI) to support academic writing. Research into ways of harnessing generative AI tools to support the academic writing process evolves so quickly that any exploration of specific software is out of date before it can be disseminated (Mustafa et al. 2024; Picasso et al. 2024). However, recent scholarship offers a principle to inform doctoral writers' exploitation of Large Language Models (LLMs) and chatbots. Specific AI tools can be deployed at particular stages of the writing process (Kim et al. 2025). For example, some software supports the early phases of the writing process, such as generating ideas, whereas other AI products are best suited to assisting with later stages of the writing process, like editing (Puxon et al. 2024; Wang 2024).

#### 2.2.3 The Development of the Writer's Identity

##### 2.2.3.1 Emotions and Identity

The empirical literature on writing group interventions for NMAHPs suggests that participants are motivated to participate in writing groups and retreats to deal with negative emotions associated with writing, such as perceived barriers to publication (Dhakal and Tornwall 2020; D. Jackson 2009; Stone et al. 2010), lack of confidence (Badenhorst et al. 2016; Bell and Murray 2021; Brandon et al. 2015; Fergie et al. 2011; Fleming et al. 2017; D. Jackson 2009; Sanderson et al. 2012; Stone et al. 2010; Wilson et al. 2013), and physical feelings associated with writing distress, fear, and dealing with distractions (Harne‐Britner and Leaver 2023). R. Murray (2012) considers the impact of writing group interventions on emotional containment. Some authors suggest that exposing the recursive nature of the writing process to a writer may cause distress (Dysthe et al. 2006; Wittman et al. 2008).

Reported writing group interventions for graduate nurses and healthcare professionals appear to associate the emotional aspects of writing development with other constructs such as developing a writing identity. In developing conceptual and operational definitions, we have found a dearth of theoretical literature on the emotional dimensions of becoming an NMAHP writer and the relationship with participating in a writing group intervention. We argue for a sociocultural framework that examines human development within cultural and social contexts, focusing on transitions in self‐identity and uncertainty. Influenced by Vygotsky's (1997) theories, this framework highlights that growth occurs through interactions with the environment, including structured experiences like writing group interventions. Zittoun (2006) offers a sociocultural framework that is key for analyzing individual transitions. Concepts like ‘rupture’ and ‘uncertainty’ describe how individuals may reassess their actions when faced with change. New writing tasks can trigger these ruptures, particularly if individuals feel ill‐equipped emotionally or cognitively. Zittoun outlines a process where ruptures lead to uncertainty, ultimately enabling transitions to new stability through psychological work.

Lee and Boud (2003) argue that identity and productivity hinge on recognising the emotional aspects of writing. This notion is supported by Sword's (2019) focus on the positive dimensions of writing, which, through our informal evaluation of writing interventions for healthcare doctoral writers, suggests that participants often report increased enjoyment in their writing development as their doctoral journey unfolds. Vincent et al. (2023) claim that writing group interventions promote pleasure, skill, and productivity, although evidence comparing enjoyment levels in group versus solo writing is lacking. They define enjoyment as a focused experience that allows doctoral students to effectively articulate their ideas, suggesting that group participation may enhance productivity and well‐being in the doctoral journey.

##### 2.2.3.2 Writing Identity and Positioning

A small selection of qualitative studies specifically considers the positioning and negotiating of a professional role and identity concerning the research writing community (Arrington et al. 2020; Fergie et al. 2011; Wittman et al. 2008) and the transitions required for future academic roles (Guerin et al. 2013). Participating in a writing group allows individuals to develop new knowledge and skills within a social context, leading to peer validation and a strengthened identity as writers.

To understand developing as an academic writer, we argue in favour of Burgess and Ivanič's (2010) work as a framework to conceptualise doctoral writers' identities in the NMAHP disciplines. Their concerns are how writers develop their identities, focusing on mature adult learners, and secondly, a discourse perspective that concerns how language constructs identity during the writing process. We adhere to their principle that the ‘authorial‐self’ demonstrates the strength of an asserted position, the extent of authorship stamped on the text, and the authoritativeness conveyed to the reader.

Ivanič's ‘discourse’ concept foregrounds a concern with social issues in language study, sitting comfortably with Gere's (1987) notion of writing groups as spaces to unite writers and audiences. It considers how writers express their emotions, for example, feeling a sense of outsiderness, which in turn can be shaped by the sociocultural context. From our earlier research on writing group interventions for nurses (Waite 2021), the intervention was a space where participants developed their identity and self‐conception to contribute to nursing knowledge through their authorship.

Intertextuality is a further component associated with developing writing identity. Ivanič (1998) argues that intertextuality requires writers to echo and align themselves with other voices. Several authors (Boote and Beile 2005; Chen et al. 2016; Kamler and Thomson 2008) note this intertextuality challenge for graduate writers. Composing a doctoral thesis requires the writer to master intertextuality and assert their voice to demonstrate a contribution to knowledge in the discipline. This suggests that the identity transitions that doctoral writers make as they develop and assert their contribution through writing can be considered.

A doctoral writer needs to internalise and externalise the full range of disciplinary concepts within appropriate genres. There is a debate in the writing literature about whether genre is amenable to formal instruction. Russell (2009) contends that genre needs to be learned but cannot be instructed because genre goes beyond a set of formal features. Transparency is required for novices to understand how established writers use the tools of the genre, enabling a stage of genre recognition. On the other hand, a ‘writing in the disciplines’ (Deane and O'Neill 2011; Wingate 2011) perspective argues that genre can and should be explicitly taught. Given the healthcare discipline's need for access to scholarly conversations, disciplinary codes and knowledge structures require more explicitness for writers by providing tools to act on the world through texts. Moreover, if doctoral writers are disseminating their work through conference abstracts and journal manuscripts, they must master authorship of different text types or genres.

#### 2.2.4 The Social Dimensions of Writing Development

The literature demonstrates that writing group interventions for NMAHP doctoral writers foster scholarly communities and collaborative practices, highlighting the social nature of writing, which needs to be introduced to novice academic writers. These interventions provide access to writing expertise (Dwyer et al. 2015; D. Jackson 2009; Rickard et al. 2009), promote the visibility of active writers (Johnson et al. 2017; MacLeod et al. 2012), facilitate dialogue about texts (Dwyer et al. 2015; Rickard et al. 2009), including peer review for constructive feedback and revising texts (Brandon et al. 2015; Dyshe 2012; Fergie et al. 2011; Johnson et al. 2017; Kulage et al. 2022; MacLeod et al. 2012). Writing groups and retreats are reported to promote collaborative practices, such as networking within an academic community and co‐authoring with other writers (Dwyer et al. 2015; Fleming et al. 2017; Guerin et al. 2013; Henshall and Lewin 2022). Furthermore, writing groups and retreats are reported to develop trust and mutual support with other writers (Garside et al. 2015; Wittman et al. 2008).

The social settings for writing group interventions for NMAHPs intersect with clinical practice areas and higher education institutions. Since the pandemic, online writing group interventions have been reported (Sun et al. 2019). It is contended that the online setting promotes international collaboration between graduate writers and other academics. Gere (1987) integrates Vygotsky's (1997) and Bakhtin's (1981) theories to conceptualise peer response dynamics in writing groups, emphasising the connection between writers and society. Vygotskian theory positions peer response as central to language development, where interactions in writing groups influence how individuals internalise and apply their learning.

Peer review is a quality process in academic writing, providing insights into publication standards and enhancing understanding of writing. Doncliff (2016) argues that peer review is valuable for graduate writers as a developmental tool because, by becoming peer reviewers, they facilitate an understanding of the rigour required for publishing (arguably, a thesis needs to be of a publishable standard), view diverse writing styles, and comprehend what is negative or positive about them concerning academic publication.

By engaging in peer reviews within writing groups, writers can clarify misconceptions, shift from solitary to public writing, enhance understanding of the process, and foster collegiality. However, feedback effectiveness can diminish if writers perceive lower proficiency among their reviewers (Ciampa and Wolfe 2023). Writers in groups may navigate their sociocultural contexts, with external factors impacting their transitions. Challenges from interpersonal relationships and disciplinary expectations can arise, but participation in writing groups can lead to active agency and contribute to the writing development of others.

#### 2.2.5 Relational Agency

The development of writer's self‐efficacy is an apparent theme in the empirical literature on writing group interventions for NMAHP doctoral students. Many studies cite Boice (1987) as a foundational pedagogical framework (MacLeod et al. 2012; Murray 2006, 2012; Murray et al. 2008; Murray and Newton 2009). Boice developed higher education writing interventions for academic staff. Boice's strategy was a staged writing process, including free and generative writing, to develop the writer's voice while raising self‐awareness of the process within social interaction with others: exploring productivity, goal setting, and giving and receiving feedback. Theoretically, it leads to self‐efficacy through behaviour change, productivity, flow, self‐understanding, and transformation of the individual's writing process.

Productivity in writing shares an implicit relationship with the writing process. Pare (2014) identifies the origin of the word ‘process’ as applied to writing development. It emerged from US composition studies inspired by an interest in how writers went from a blank page to a complete text. Very often, it starts with goal setting. The practice of goal setting is claimed to enhance productivity and meet deadlines. It can also help reduce stresses arising from extended or complex writing projects. Tosi et al. (1991) posit that specific, challenging goals lead to higher performance than vague or easy goals. They argue that writers who set clear, measurable goals are likelier to complete their projects efficiently and on time. Self‐efficacy plays a significant role in academic writing goal setting. Some studies report the relationship between behaviour change and its influence on outputs following a writing group intervention (Johnson et al. 2017; MacLeod et al. 2012; Murray and Newton 2009) underpinned by Bandura's (1977) social learning theory, which concerns self‐regulatory capacities and environmental inducements through cognitive support. By observing others, people can exercise some control over their behaviour, leading to self‐generated behaviour patterns. Bandura's perspective may provide an appropriate theory to explain such outcomes. However, the focus is on individual behaviour, and other theoretical lenses may tell us more about how resources from writing group interventions are used to propel writers forward. Writing development is complex, involving cognitive, social, and emotional dimensions.

Instead of self‐efficacy, we advocate for the use of Edwards' (2005) theory of relational agency, which is underpinned by Edwards' (2011) cultural‐historical concept of ‘what matters.’ Rooted in Leontiev's (1981) idea of the interplay between individuals and society, this theory emphasizes how individuals reshape the object of activity through a shared understanding of ‘what matters.’ This collaborative understanding can be seen in writing group interventions, where participants adjust their approaches by collectively identifying ‘what matters’ and deepening their knowledge of writing as a complex activity.

However, people do not bracket themselves once they join a writing group intervention. They bring motives and values linked to their societal practices, activities, and actions (Edwards 2011), influencing ‘what matters’. A writing group intervention for NMAHP writers will usually include the expectations and norms of academic supervisors and the practice and disciplinary expectations of becoming a clinical academic, including disseminating writing to improve patient care. Edwards recommends that researchers in the tradition identify the potential for misalignment with what society demands in interplay with what matters. The critical issue for the researcher is to reveal those differences to analyze how individuals negotiate them.

It is challenging to balance the competing demands of clinical work and maintaining regular scholarly writing habits, illustrating the misalignment between demand and what matters. However, some authors (Johnson et al. 2017; Sword 2019) contest the productivity mantra, claiming that writing regularly may not be possible or desirable and that focusing on productivity minimizes writing development's social, artisanal, and emotional aspects.

## 3 Data Sources

### 3.1 Methods

#### 3.1.1 Item and Measurement Development

##### 3.1.1.1 Available Measures

To determine if any similar instrument could be modified and used to assess the constructs underpinning DAWNMAHP, we drew on our literature review on writing group interventions for NMAHPS at the graduate level. Only Harne‐Britner and Leaver (2023) reported using and adapting a validated tool. They modified the post‐secondary writerly self efficacy scale (PSWSES) (Schmidt and Alexander 2012) to create the RNP‐PSWSES for registered nurses who have completed research projects and are undertaking a mentored writing group for publication intervention. The adapted 20‐item scale focused on self efficacy variables such as self‐perceived mastery, vicarious learning, reduced stress reaction and negative emotions associated with writing processes and asked respondents to rate personal effectiveness as academic writers using a percentage scale from zero to one hundred. Harne‐Britner and Leaver found acceptable reliability with pre‐test Cronbach's ∝ = 0.728 and excellent reliability with post‐test ∝ = 0.818, suggesting a statistically significant difference between the pretest and post‐test scores (*z* = −1.96, *p* = 0.050). However, the participant sample was small (*n* = 10).

Items in the RNP‐PWESES may be mapped onto time management and awareness of the writing process constructs. However, we did not adopt the RNP‐PSWSES as a whole as a measure for DAWNMAHP because it was designed to be administered to individuals attending undergraduate tutorials at North American Writing Centres and lacks items to consider: the social, identity, and agency domains of doctoral writing development that relate to writing group interventions for NMAHPs.

Outside of the NMAHP disciplines, Astaneh, Abdullah, et al. (2024), Astaneh, Raeisi Shahraki, et al. (2024) developed and validated a tool to measure confidence in medical academic writers participating in writing group interventions. They identified and piloted 18 items concerning ‘confidence in the use of appropriate academic English language required for publishing articles in peer‐reviewed medical journals.’ Participants were given a 5‐point Likert Scale response option ranging from 1 (no confidence) to 5 (extremely confident). Thirty‐five participants who had previously participated in a writing group intervention facilitated by the researchers responded. The tool demonstrated a content validity index (CVI) of 0.75 and convergent validity of 0.79, suggesting acceptable validity. A factor analysis demonstrated unidimensionality, confirming that the items measure the same property. A strength of this research is the fine‐grained analysis of individuals’ self‐assessment of their proficiency in producing academic publications. However, the items in the survey created by Astaneh, Abdullah, et al. (2024), Astaneh, Raeisi Shahraki, et al. (2024) concentrate on the knowledge required for composing a journal manuscript (product). Therefore, we chose not to use items from this tool due to its limited applicability to the various aspects of doctoral writing development.

McLellan et al. (2024) adapted Zimmerman and Bandura's (1994) validated 11‐item self‐efficacy scale to measure self‐efficacy in academic writing concerning cross‐disciplinary early career researchers (ECRs) participating in structured writing retreats. Participants were given a 5‐point Likert Scale Option ranging from 1 (the poorest) to 5 (the greatest). The questionnaire was administered before participating in the retreats (baseline) and following the final retreat (post‐measure). The final sample size comprised 26 participants. All responses improved from baseline, and seven statements showed significant improvements (all *p <* 0.05). These included self‐efficacy in starting to write, setting goals, dealing with distractions, revising drafts for coherence and conciseness, sharing drafts for feedback, and regularly completing writing goals. The emphasis on self‐efficacy is connected to several constructs we have identified regarding developing doctoral writing for NMAHPs participating in writing group interventions. However, we did not utilize items from the existing tool because it lacks comprehensiveness in the domains we have identified for doctoral writing development.

From this analysis of the strengths and limitations of existing research instruments designed to measure academic writers' development, we conclude that more comprehensive scale development is needed to consider the complex, time‐consuming, and formative process of becoming a doctoral writer in the NMAHP disciplines. Furthermore, this investigates how this relates to participation in writing group interventions. This study investigates the relationship between writing group interventions and doctoral and academic writing development for the NMAHP disciplines based on the writer's self‐assessment. Our goal is to develop an instrument rigorously grounded in the existing literature whilst improving upon previous scale limitations through a more comprehensive inclusion of items and a valid and reliable instrument that may be administered to measure writing development in NMAHP doctoral writing group interventions.

#### 3.1.2 Item Definition and Identification

As a first step, we generated a pool of items based on the empirical evidence (Almanasreh et al. 2019) from the literature review we undertook to develop conceptual and operational definitions. We included four items from the DNP‐PWESES (Harne‐Britner and Leaver 2023) related to time management and awareness of the writing process. We also drew on our experience of facilitating writing group interventions for NMAHP doctoral candidates and early career professionals (ECRs) to support thesis composition and writing for publication. We collected feedback from participants regarding which aspects of writing group interventions were effective and what areas could be improved.

In addition, we consulted with key stakeholders, such as academic colleagues who supervise NMAHP doctoral students and support their writing development, current doctoral students, and alumni. Our approach identified 39 items (Table 1) across five domains: time management, awareness of the writing process, the development of the writer's identity, the social dimensions of writing development, and relational agency.

**TABLE 1:** Construct validity index.

Item	CVI
1. What is your current work/academic role?	0.73
2. How long have you been in your current role?	0.53
3. What is your first language?	0.87
4. Do you speak any additional languages?	0.67
5. Do you have a specific learning difference (SPLD)?	0.80
6. What type of paper are you currently writing?	1.00
7. What is your writing‐for‐publication history?	0.93
8. I can manage my time when faced with a doctoral writing task	0.87
9. When I have a doctoral writing task, I create deadlines for myself	0.87
10. I value the protected writing time during a writing group, retreat or workshop	0.93
12. I can develop my ideas through the process of doctoral writing	0.73
13. I can find and correct my grammatical errors in my doctoral writing	1.00
14. I can improve the flow of argument and readability of my doctoral writing	1.00
15. I can develop my writer's voice by revising my draft doctoral writing	0.87
16. I can find and incorporate appropriate evidence to support important points in my doctoral writing	0.93
17. When I read a rough draft, I can identify gaps when they are present in my doctoral writing	0.87
18. Once I have completed a draft, I can revise my doctoral writing to signpost the reader	0.73
19. I have a sense of the target audience for my doctoral writing	0.93
20. Participating in a writing group, retreat, or workshop has raised my awareness of the doctoral writing process	0.80
21. Participating in a writing group, retreat, or workshop has raised my awareness of doctoral writing as a product	0.60
22. I know how to produce different writing genres required by the nursing/allied health professional doctoral field	0.80
23. Participating in a writing group/retreat supports my writing to demonstrate knowledge and expertise in the field	0.80
24. During the doctoral writing process, I will likely seek informal feedback from a peer, colleague, friend, or family member.	1.00
25. I can give them valuable feedback when I read drafts written by doctoral peers	0.87
26. I will likely use informal and formal feedback to revise my doctoral writing	0.93
27. I can transition from writing individually to co‐authoring with others on journal papers during the doctoral writing process	0.87
28. Being in a space with others during a writing group, retreat, or workshop encourages me to write	0.80
29. Talking with others during a writing group, retreat, or workshop encourages me to write	0.67
30. Talking with others during a writing group, retreat, or workshop helps me understand my doctoral writing process	0.73
31. Participating in a writing group, retreat or workshop helps me build relationships with others and understand my contribution as a doctoral writer	0.73
32. I can undertake doctoral writing without negative feelings such as stress, anxiety, fear or distress	0.60
33. When I undertake doctoral writing, I have positive feelings such as motivation, happiness and excitement	0.67
34. I believe in myself as a doctoral writer	0.80
35. Exposure to the writing process during a writing group, retreat or workshop makes me anxious.	0.73
36. Participating in a writing group, retreat or workshop motivates me to write.	0.73
37. Participating in a writing group, retreat or workshop enables me to enjoy writing	0.67
38. Participating in a writing group, retreat or workshop enables me to believe in myself as a doctoral writer	0.53
39. Participating in a writing group, retreat or workshop helps me build relationships with others and understand my contribution as a doctoral writer	0.73

#### 3.1.3 Judgement‐Quantification Stage

As a second step, an early draft of the survey questionnaire was distributed to a panel of NMAHP writing experts, including editors and academics from an international context with longstanding experience in NMAHP doctoral education (Table 1).

#### 3.1.4 Instrument Testing

As a third step, we created a Qualtrics form to capture the final five domains and items following the expert validation stage. Responses are measured on a 7‐point Likert scale from 1 (strongly disagree) to 7 (strongly agree). We also included open questions concerning the use of AI and an open question for each domain to gather optional participant feedback, leading to a total of 44 items.

We approached current applicants and previous participants for the institutional and cross‐institutional writing group interventions we offer NMAHPs. The former participants only included those previously interested in our research concerning academic writing development. To test the instrument's reliability and validity, we obtained ethical approval from the Oxford Brookes University research ethics committee on 17 January 2025 to recruit a representative sample of participants (Study No. L25359). We contacted them, explained the study's purpose, and gained their consent to participate while ensuring anonymity and confidentiality.

#### 3.1.5 Data Analysis

Before statistical analysis, all demographic and open‐answer questions were excluded, and the dataset was checked for missing values and outliers, with no major issues detected. A Principal Component Analysis (PCA) with Varimax rotation was conducted to assess the underlying structure of the survey. Given the small sample size (*n* = 36), PCA was chosen over Exploratory Factor Analysis (EFA) to maximize factor interpretability and stability.

The factorability of the data was assessed through the Kaiser‐Meyer‐Olkin (KMO) Measure of Sampling Adequacy and Bartlett's Test of Sphericity. Factors were extracted by examining the Scree Plot for the ‘elbow’ and applying Kaiser's criterion (Eigenvalues > 1). Items with communalities below 0.50 were removed to improve factor loadings and overall model fit. A factor loading cutoff of 0.50 was applied to determine which items were retained in the final factor solution. Following this, the survey included 25 items measuring 5 domains, yielding a relatively high item‐to‐domain ratio. According to MacCallum et al. (1999), such overdetermination allows confidence in the results even when based on a comparatively smaller sample size. To assess internal consistency, Cronbach's Alpha was calculated for each extracted factor, with values above 0.70 considered acceptable. All statistical analysis was conducted using IBM SPSS Statistics (version 28.0).

## 4 Findings

### 4.1 Content Validation

We received 15 responses, exceeding the recommended minimum range of six to ten experts for content validation (Almanasreh et al. 2019) from across the international expert NMAHP writing community. Participants rated each proposed survey item on a four‐point Likert scale, with response categories: ‘Not Relevant’, ‘Somewhat Relevant’, ‘Quite Relevant’, and ‘Highly Relevant’. The content validity index CVI was used to assess the acceptability of each item by calculating the proportion of respondents who rated the item as ‘Quite Relevant’ or ‘Highly Relevant’ (Lynn 1986). The minimum threshold of 0.78 for each item to be included was used to decide acceptability (Polit et al. 2007). The results are presented in Table 1.

Additionally, the CVI/AVE was calculated to assess the overall validity of the scale, and this yielded a score of 0.8, below the acceptable threshold of 0.9 (Polit et al. 2007). However, when recalculated using only the accepted items, the score met the required validity standard (= 0.9). Therefore, refining the highlighted items above to align with the rest of the questionnaire should ensure both item‐level and overall scale validity.

Additionally, the experts were invited to provide qualitative feedback on the items' face validity. While publication history scored 0.93 on CVI, respondents identified it as vague. They suggested that asking about the current writing task and previous outputs concerning text type would be more relevant. The length of time in the current role and additional languages spoken were deemed irrelevant, corresponding with the CVI scores. Consequently, we amended the demographic items. Items that scored lower on CVI referred to writing group intervention participation and were the most commented on by participants. Importantly, we received feedback that some items included leading questions about taking part in writing groups and retreats and were unlikely to capture non‐positive aspects of participating in such group‐based events or could be seen as devaluing writing as an independent activity. As a result, we decided to remove all references to writing group interventions from the items.

Some participants suggested that aspects such as grammar and punctuation and the increasing use of writing software and AI composition tools should be considered when assessing the writing process. Given the significance of this trend, we added three items to address these considerations. The items relating to the concept of writing identity were also commented upon. Our expert consultants suggested that doctoral writers would not understand terms such as ‘genre’ and ‘demonstrating knowledge and expertise in the field’, which required unpacking into simpler terms. For example, in lieu of genre, we asked the respondents to identify the type of paper they were currently writing (e.g., essay, thesis chapter, journal manuscript). Instead of ‘expertise’, we asked respondents about their experience of academic and provided a list of different text types. We expanded and simplified these items from two to six. Once we had revised the overall items, considering expert validation, 44 items remained (See File S1).

### 4.2 Instrument Reliability and Construct Validity

The revised pool of items was tested with 36 participants who were eligible to participate in the study. They were either an early career researcher (ECR), enrolled in a doctoral programme, or designated doctoral pre‐programme in nursing, midwifery, allied health, and social care. These included 22 postgraduate students, seven ECRs, and seven pre‐doctoral candidates. Each participant had attended at least one writing retreat or group and completed the DAWNMAHP survey tool between 24th February 2025 and 22nd March 2025.

Table 2 presents the participants' demographic information, indicating that doctoral and early‐career researchers were engaged in composing multiple academic research writing genres.

**TABLE 2:** Participants' demographic information.

Type of writer		Current text				
		Grant proposal	Journal manuscript	Thesis chapter	Essay	Other
Pre‐doctoral	7	3	1			3
Doctoral	22	1	9	21	1	
Early Career researcher	7	3	7			

### 4.3 Factor Analysis

The KMO value was 0.579, which is considered moderate but acceptable sampling sufficiency for PCA. Bartlett's Test of Sphericity was significant (*χ* ^2^ = 608.81, *p* < 0.001), indicating that the data were sufficiently correlated for factor extraction. After reviewing the Scree Plot and the total variance explained, a final solution with five factors was retained, accounting for 69.45% of the total variance. Items with low communalities (< 0.50) were removed, which improved factor clarity. This included five items (File S1) 5, 10, 16, 18 and 30. When factor loading, one question (12) did not load onto any factor and was consequently removed from further analysis.

The rotated component matrix showed that all retained items loaded strongly (≥ 0.50) onto their respective factors, with no significant cross‐loadings. Table 3 shows the factors that were identified from the items as to what they were likely measuring.

**TABLE 3:** Factor loadings.

	Factor loading
*Factor 1: Time management*	
I can effectively manage my time when faced with a writing task	0.82
I have developed strategies to address competing demands (clinical work, personal responsibilities) while maintaining writing time	0.85
I can create deadlines for myself when I have a writing task	0.73
*Factor 2: Awareness of the writing process*	
I write regularly	0.60
I can find and correct grammatical errors in my writing	0.85
I understand how to improve the clarity of my argument in my writing	0.85
I can develop my writer's voice by revising my draft writing	0.81
When I read a rough draft, I can identify gaps when they are present in my writing	0.67
When I read a rough draft, I can identify gaps when they are present in my writing	0.67
I understand how to refine my writing to guide potential readers	0.61
*Factor 3: The development of the writer's identity*	
I will likely use informal and formal feedback to revise my writing	0.61
I can move between individual thesis writing and co‐authoring journal papers during the writing process	0.58
Understanding my contribution strengthens my sense of identity as a writer	0.71
Disseminating my ideas strengthens my sense of identity as a writer	0.79
I contribute to knowledge through my writing	0.72
*Factor 4: The social dimensions of writing development*	
I feel anxious when writing in the company of other writers	0.76
I feel motivated when writing in the company of other writers	0.86
I enjoy writing as a solitary activity	0.68
I can give valuable feedback if I read drafts written by peers	0.73
Being in a space with other writers encourages me to write	0.65
*Factor 5: Relational agency*	
I can undertake writing without negative feelings such as stress, anxiety, fear or distress	0.65
I gain an understanding of my writing processes through discussions with other writers	0.66
Talking with others about my writing encourages me to write	0.70
I am confident in writing different outputs and text types	0.58
I know how to position my academic voice alongside other authors	0.53

Factor	No. of items	Cronbach's alpha
Factor 1: Time management	3	0.79
Factor 2: Awareness of the writing process	6	0.87
Factor 3: The development of writer's identity	6	0.82
Factor 4: The social dimensions of writing development	5	0.81
Factor 5: Relational agency	5	0.72

### 4.4 Factor Correlations

The five factors identified were checked using Pearson's correlation coefficient to provide more insight into the factor structure. Awareness of the writing process, relational agency, and time management showed moderate correlations (ranging from *r* = 0.30 to *r* = 0.46), suggesting that these factors are related but distinct aspects of writing behaviour. Awareness of the writing process is also correlated with writing identity (*r* = 0.31), indicating a moderate but distinct link between the writing process and the sense of identity as a writer. The social dimensions of writing development demonstrated low correlations with all other factors (ranging from *r* = −0.02 to *r* = 0.24), which may reflect its unique role in the context of writing groups. Despite these low correlations regarding the social dimension factor, this highlights the importance of having a survey tool with a section dedicated to this aspect, as it appears not to be measured through other ways.

### 4.5 Reliability Analysis

To assess internal consistency, Cronbach's Alpha was calculated for each factor. All factors demonstrated good reliability (*α* > 0.70), indicating that the items within each factor were measuring the same underlying concept (Table 3).

## 5 Discussion

Our research aim was to design and validate the DAWNMAHP questionnaire to measure the relationship between writing group interventions and doctoral and academic writing development. We achieved this aim by generating five factors with internal content validity.

### 5.1 Reliability

Following the removal of items with communalities below 0.50 to improve factor loadings and overall model fit, the pilot yielded data sufficiently correlated for factor extraction, generating five factors and ensuring that only items with strong loadings are included in the analysis. To ensure internal consistency, the reliability of factors was evaluated using Cronbach's Alpha. All five factors demonstrated good reliability (*α* > 0.70), indicating that each factor's items measured the same underlying concept.

### 5.2 Validity

The piloting stage of the DAWNMAHP survey tool demonstrates that PCA can be systematically applied to measure relationships between social writing intervention and doctoral and ECR research writers' development (File S1). The main implication of the validation process is that the analysis has been robust and interpretable despite the limitations posed by a relatively small sample size. Having adopted PCA to identify implicit structures within the questionnaire responses, favouring this approach over exploratory factor analysis appears justified. This choice was made to maximise factor interpretability and stability, which is crucial when working with limited data. However, this approach may be revised for future research with larger sample sizes.

Concerning the factorability of data produced in the pilot phase of research, the Kaiser‐Meyer‐Olkin measure of sampling adequacy and Bartlett's test of sphericity have been proven to be appropriate. Whilst the Kaiser‐Meyer‐Olkin measure of 0.579 can be considered moderate, this is acceptable for principal component analysis. Bartlett's test was significant (*χ* ^2^ = 608.81, *p* < 0.001), confirming that the data were sufficiently correlated for factor extraction.

### 5.3 What DAWNMAHP Measures

**FIGURE 1:**
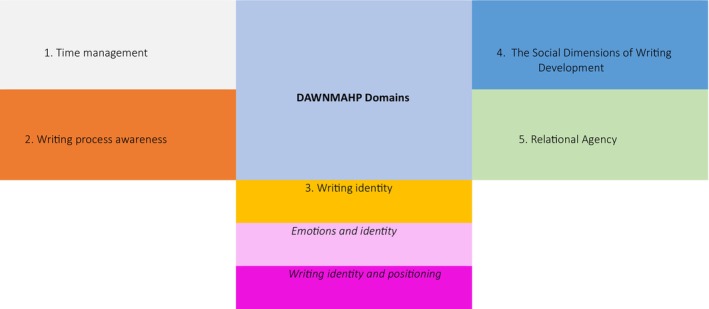
DAWNMAHP domains.

The key findings regarding the DAWNMAHP survey tool are as follows (See Figure 1):

Factor 1: Time management was the most precise result, correlating with expected items, including the ability to address competing demands and to create deadlines for lengthy or complex writing tasks.

Factor 2: Writing process awareness showed strong content validity, including regularly practising writing and revising draft texts. After removing items with communalities below 0.50 to enhance factor loadings and improve overall model fit, we were surprised to find that most eliminated items, listed in supplementary file x, were from the writing process awareness domain. This includes items such as “I can develop my ideas through the process of writing” and “writing is a long‐term iterative process.” With the latter item, some respondents commented that they did not fully understand the wording of the question coupled with the reality and time‐consuming nature of academic writing.

During the consultation stage of developing DAWNMAHP, it was not apparent that strategies to raise awareness of the doctoral writing process could potentially play a role in personal academic writing development. For example, many doctoral writers tell us that the writing processes involved in writing a thesis would benefit from more exposition and explanation. These include the relationship between writing and thinking, writing to generate ideas, steps undertaken to produce a text, the writing process and product, and making writing transitions between essay writing, thesis writing, and writing for publication. Therefore, this is another result worthy of further investigation, including rewording the item and administering it to a larger sample size.

Factor 3: Writing identity includes emotional dimensions, such as experiencing positive feelings during the doctoral writing process. This suggests a need for more research on the role of emotions in developing authorial identity. It also involves social elements like peer review and concepts identified in the literature review, such as authoring and co‐authoring various texts. Writing process awareness also correlates with writing identity (*r* = 0.31), indicating a moderate but distinct link between the writing process and the sense of identity as a writer, but distinct enough to be measured separately.

Factor 4: The results demonstrated that the social dimensions of writing development exhibited a low correlation with other factors, yet they are a crucial component of writing group interventions. Conversely, the factor also indicates the value of writing as a solitary activity in addition to writing within a social group.

Factor 5: Relational agency was a noteworthy result because this factor brought together items focused on personal awareness and agency but related to collaborative dialogue with others. This construct contrasts with some perspectives in the empirical and theoretical literature reported earlier, which tend to prefer self‐efficacy as a core construct in academic writing development (McLellan et al. 2024). These findings merit further research with a larger sample size to examine further possible connections between personal and relational agency in development as an academic writer.

### 5.4 Implications for Practice and Research

The five factors identified in the pilot study have key implications for future research. Next, we will administer the DAWNMAHP questionnaire to participants before and after they join a writing group intervention to assess changes in their self‐evaluation of writing development.

DAWNMAHP has potential for adaptation to other writing levels, for example, when a learner is confronted with a new and complex writing task, such as composing a master's dissertation. It also has the potential to measure writers' development longitudinally by administering the questionnaire over several points in time, which we plan to validate in our future work. As our consultation process with doctoral students and alumni showed, DAWNMAHP has potential as a self‐assessment tool for academic research writers.

This study focused on the NMAHP disciplines, whereas future work will explore how well the tool measures writing development for doctoral writers from other disciplines participating in similar interventions. As we argued from the outset of this paper, the lack of validated tools to measure academic writing development is a cross‐disciplinary issue.

Based on feedback from key stakeholders during the development and validation process, doctoral writers have indicated that DAWNMAHP is a valuable tool. It provides information, access to resources, and a means for students to self‐assess their writing throughout their doctoral journey. This capability enables students to identify areas that require development. DAWNMAHP can be utilized at the beginning of the supervisory process, allowing supervisors and students to understand the students' current strengths and needs.

*The* DAWNMAHP *survey tool* has the potential for translation and validation in other countries, which will enhance the international NMAHP doctoral and ECR community. For example, it could facilitate research projects to enable comparison and benchmarking. Validating DAWNMAHP in several languages and contexts could support this. Writing group interventions are popular but have resource implications. In summary, this investigation highlights the criticality of researching their benefits through valid and reliable methods to establish a sustainable NMAHP doctoral writing evidence base. This paper's primary contribution to doctoral education for NMAHPs is the design and validation of a new research tool to measure the relationship between participation in social writing interventions and the development of individual writers.

### 5.5 Limitations

A key limitation is the statistical analysis: PCA was used instead of EFA to ensure factor interpretability due to the small sample size (*n* = 36). However, it would be beneficial to replicate this analysis further with a larger sample size using EFA to confirm the construct validity and generalisability of the survey. The KMO value (0.579) was moderate but acceptable, and increasing the sample size would likely improve this value, enhancing confidence in the validity and overall robustness of DAWNMAHP.

## 6 Conclusion

This paper reported the initial development of DAWNMAHP, a novel and reliable tool designed to assess the impact of writing group interventions on individual writers' development. This research tool focuses on the key factors of: time management, writing process awareness, writing identity, social aspects of writing development, and relational agency. The DAWNMAHP survey tool has the potential for further development as a pre‐ and post‐assessment tool for doctoral writers participating in writing group interventions. This new survey tool can also be deployed to broader contexts to establish an evidence base for the benefits of writing group interventions for doctoral writers and ECRs. This methodology paper's overall contribution is a new research tool for measuring the relationship between participation in social writing interventions and the development of individual writers.

## Ethics Statement

State that the relevant fieldwork permission was obtained and list the permit numbers. Ethical approval was sought from Oxford Brookes University Research Ethics Committee, and permission to collect participant data was granted in February 2024 UREC Registration No: L25359. Dr. Heather Callagan is a lecturer in mathematics and statistics.

## Conflicts of Interest

The authors declare no conflicts of interest.

## Supporting information


**File S1:** jan70290‐sup‐0001‐FileS1.docx.

## Data Availability

Data available on request due to privacy/ethical restrictions:The data that support the findings of this study are available on request from the corresponding author. The data are not publicly available due to privacy or ethical restrictions.
