# Supplementary material for: Validating the Doctoral and Academic Writing in Nursing, Midwifery and Allied Health Profession Survey Questionnaire for Writing Group Interventions
Source: J Adv Nurs. 2025 Oct 27;82(5):4393–407. doi: 10.1111/jan.70290 (PMC13069194; doi:10.1111/jan.70290)
Supplement: Supplementary file 1 — File S1: jan70290‐sup‐0001‐FileS1.docx. [file JAN-82-4393-s001.docx]

## Items following expert validation

| **Demographic items** | |
| --- | --- |
| 1. | What is your current academic role? |
| 2. | What type of paper are you working on? (Tick all that apply)   - Essay - Dissertation or thesis chapter - Journal manuscript - Grant proposal - Other |
| 3. | What is your experience of academic writing? Tick all that apply   - Undergraduate degree assessments - Master’s dissertation or capstone project - Conference abstract - Journal manuscript - Doctoral thesis - Research grant proposal - Creative writing/story/poem - Other |
| 4 | Please comment on the above questions |

**Time management**

| **Indicate the extent to which you agree or disagree with the following statements.** | |
| --- | --- |
| 5. | I can effectively manage my time when faced with a writing task. |
| 6. | I have developed strategies to address competing demands (clinical work, personal responsibilities) while maintaining writing time. |
| 7. | I can create deadlines for myself when I have a writing task. |
| 8. | I value protected time for my writing. |
| 9. | I write regularly. |
| 10. | **Open text question**: Do you use any strategies to ensure that you progress with your writing regarding time management and productivity? |
| 11. | Please comment on the above questions |

**Awareness of the writing process**

| **Indicate the extent to which you agree or disagree with the following statements.** | |
| --- | --- |
| 12. | Writing is a long-term recursive process. |
| 13. | I can develop my ideas through the process of writing. |
| 14. | I can find and correct grammatical errors in my writing. |
| 15. | I understand how to improve the clarity of my argument for my writing. |
| 16. | I can develop my writer’s voice by revising my draft writing. |
| 17. | I can find and incorporate appropriate evidence to support my writing. |
| 18. | When I read a rough draft, I can identify gaps when they are present in my writing. |
| 19. | I keep in mind my target audience when writing. |
| 20. | I understand my writing processes through discussions with other writers. |
| 21. | I understand how to refine my writing to guide potential readers. |
| 22. | **Yes or No**: I use generative artificial intelligence tools while writing. |
| 23. | **If you answered yes to the previous question**, List the generative artificial intelligence tools you use during the writing process and indicate how you use them. |

**The development of the writer’s identity**

| **Indicate the extent to which you agree or disagree with the following statements.** | |
| --- | --- |
| 24. | Understanding my contribution strengthens my sense of identity as a writer. |
| 25. | Disseminating my ideas strengthens my sense of identity as a writer. |
| 26. | I contribute to knowledge through my writing. |
| 27. | I am confident in writing different outputs and text types. |
| 28. | I know how to find and position my academic voice alongside other authors. |
| 29. | Please comment on the above questions. |

**The social dimensions of writing development**

| **Indicate the extent to which you agree or disagree with the following statements.** | |
| --- | --- |
| 30. | I gain an understanding of my writing process through discussions with other writers. |
| 31. | I will likely seek informal feedback from a peer, colleague, friend, or family member during the writing process. |
| 32. | I can give them valuable feedback if I read drafts written by peers. |
| 33. | I will likely use informal and formal feedback to revise my writing. |
| 34. | I can move between writing individual thesis writing and co-authoring journal papers during the writing process. |
| 35. | Being in a space with other writers encourages me to write. |
| 36. | Talking with others about my writing encourages me to write. |
| 37. | Please comment on the above questions. |

**Relational agency**

| **Indicate the extent to which you agree or disagree with the following statements.** | |
| --- | --- |
| 39. | I can undertake writing without negative feelings such as stress, anxiety, fear or distress. |
| 40. | When I undertake writing, I have positive feelings such as motivation, happiness and excitement. |
| 41. | I feel anxious when writing in the company of other writers. |
| 42. | I feel motivated when writing in the company of other writers. |
| 43. | I enjoy writing as a solitary activity. |
| 44 | Please comment on the above questions |

## Items following factor correlations

Factor 1 - Time Management

1. I can effectively manage my time when faced with a writing task.
2. I have developed strategies to address competing demands (clinical work, personal responsibilities) while maintaining writing time.
3. I can create deadlines for myself when I have a writing task.

Factor 2 - Writing Process Awareness

1. I write regularly. Indicate the extent to which you disagree or agree with the statement.
2. I can find and correct grammatical errors in my writing. Indicate the extent to which you disagree or agree with this statement.
3. I understand how to improve the clarity of my argument for my writing. Indicate the extent to which you disagree or agree with this statement.
4. I can develop my writer's voice by revising my draft writing. Indicate the extent to which you disagree or agree with this statement.
5. When I read a rough draft, I can identify gaps when they are present in my writing. Indicate the extent to which you disagree or agree with this statement.
6. I understand how to refine my writing to guide potential readers. Indicate the extent to which you disagree or agree with this statement.

Factor 3 The development of writing identity

1. When I undertake writing, I have positive feelings such as motivation, happiness and excitement. Indicate the extent to which you disagree or agree with this statement.
2. I will likely use informal and formal feedback to revise my writing. Indicate the extent to which you disagree or agree with this statement.
3. I can move between individual thesis writing and co-authoring journal papers during the writing process. Indicate the extent to which you disagree or agree with this statement
4. Understanding my contribution strengthens my sense of identity as a writer. Indicate the extent to which you disagree or agree with this statement.
5. Disseminating my ideas strengthens my sense of identity as a writer. Indicate the extent to which you disagree or agree with this statement.
6. I contribute to knowledge through my writing. Indicate the extent to which you disagree or agree with this statement.

Factor 4 - The social dimensions of writing development

1. I feel anxious when writing in the company of other writers. Indicate the extent to which you disagree or agree with this statement.
2. I feel motivated when writing in the company of other writers. Indicate the extent to which you disagree or agree with this statement.
3. I enjoy writing as a solitary activity. Indicate the extent to which you disagree or agree with this statement.
4. I can give valuable feedback if I read drafts written by peers. Indicate the extent to which you disagree or agree with this statement.
5. Being in a space with other writers encourages me to write. Indicate the extent to which you disagree or agree with this statement.

Factor 5 -Relational Agency

1. I can undertake writing without negative feelings such as stress, anxiety, fear or distress. Indicate the extent to which you disagree or agree with this statement.
2. I gain an understanding of my writing processes through discussions with other writers. Indicate the extent to which you disagree or agree with this statement.
3. Talking with others about my writing encourages me to write. Indicate the extent to which you disagree or agree with this statement.
4. I am confident in writing different outputs and text types. Indicate the extent to which you disagree or agree with this statement.
5. I know how to position my academic voice alongside other authors. Indicate the extent to which you disagree or agree with this statement.

Removed items with communalities below 0.50

- Writing is a long-term iterative process.
- I value protected time for my writing. Indicate the extent to which you disagree or agree with the statement.
- I can develop my ideas through the process of writing. Indicate the extent to which you disagree or agree with the statement.
- I can find and incorporate appropriate evidence to support my writing. Indicate the extent to which you disagree or agree with this statement.
- I keep in mind my target audience when writing.  Indicate the extent to which you disagree or agree with this statement.
- I will likely seek informal feedback from a peer, colleague, friend, or family member during the writing process. Indicate the extent to which you disagree or agree with this statement.
